# The tumor-nerve circuit in breast cancer

**DOI:** 10.1007/s10555-023-10095-1

**Published:** 2023-03-31

**Authors:** Qiuxia Cui, Dewei Jiang, Yuanqi Zhang, Ceshi Chen

**Affiliations:** 1 Affiliated Hospital of Guangdong Medical University Science & Technology of China, Zhanjiang, 524000 China; 2 Department of Breast Surgical Oncology, National Cancer Center/National Clinical Research Center for Cancer/Cancer Hospital and Shenzhen Hospital, Chinese Academy of Medical Sciences and Peking Union Medical College, Shenzhen, 518116 China; 3 Key Laboratory of Animal Models and Human Disease Mechanisms of the Chinese Academy of Sciences and Yunnan Province, Kunming Institute of Zoology, Chinese Academy of Sciences, Kunming, 650201 China; 4 Academy of Biomedical Engineering, Kunming Medical University, Kunming, 650500 China; 5 The Third Affiliated Hospital, Kunming Medical University, Kunming, 650118 China

**Keywords:** Innervation, Breast cancer, Neurotransmitter, Psychological stress, β-blockers

## Abstract

It is well established that innervation is one of the updated hallmarks of cancer and that psychological stress promotes the initiation and progression of cancer. The breast tumor environment includes not only fibroblasts, adipocytes, endothelial cells, and lymphocytes but also neurons, which is increasingly discovered important in breast cancer progression. Peripheral nerves, especially sympathetic, parasympathetic, and sensory nerves, have been reported to play important but different roles in breast cancer. However, their roles in the breast cancer progression and treatment are still controversial. In addition, the brain is one of the favorite sites of breast cancer metastasis. In this review, we first summarize the innervation of breast cancer and its mechanism in regulating cancer growth and metastasis. Next, we summarize the neural-related molecular markers in breast cancer diagnosis and treatment. In addition, we review drugs and emerging technologies used to block the interactions between nerves and breast cancer. Finally, we discuss future research directions in this field. In conclusion, the further research in breast cancer and its interactions with innervated neurons or neurotransmitters is promising in the clinical management of breast cancer.

## Introduction

Breast cancer is currently one of the leading cancer types worldwide [1, 2]. Despite the improvement in therapeutic strategies, approximately 35% of patients experience recurrence, metastasis, and drug resistance [3]. Neuro-oncology plays important roles in the progression of various tumors, including breast cancer [4]. Approximately 20% of cancer patients eventually develop brain metastases [5, 6]. In tumor microenvironment, innervation and perineural invasion have recently been identified as one of the latest hallmarks of tumors [7]. Tumor innervation refers to breast tumor parenchyma infiltrated by sympathetic, parasympathetic, and sensory nerves and perineural invasion refers to tumor cell growth and invasion along the neural fibers. A study of 369 breast cancer patients with primary invasive ductal carcinoma showed that 28% of cases had nerve invasion, according to the isolated nerve fibers or axons [8].

Nerves within tumors, also called perineural infiltration or innervation, play an important role in solid and hematological malignancies [9–11]. In the solid tumor microenvironment, nerve fibers usually originate from the peripheral nervous system [12, 13]. Tumor innervation is associated with the progression of multiple tumors [14], including breast cancer [15, 16]. It has been demonstrated that nerves promote breast cancer tumorigenesis and that innervation is associated with a poor prognosis (overall and disease-free survival) [17].

The innervation of tumors is mainly derived from the autonomic nervous system, including sympathetic, parasympathetic, and sensory nerves. It is well known that the sympathetic and parasympathetic/vagal nerves control the function of almost any organ, maintaining systemic homeostasis under physiological conditions [18]. The autonomic nervous system plays an important role in the regulation of cancer cell proliferation, differentiation, apoptosis, migration, and invasion [19].

In this review, we focus mainly on the innervation of breast cancer, aiming to review the role and functional mechanisms of nerves in breast cancer progression and brain metastasis. A more comprehensive understanding of the role of innervations in breast tumors will provide promising therapeutic strategies for breast cancer.

## Nerve distribution in the breast (cancer)

### Nerve system in normal breast tissues

The human breast is a modified cutaneous exocrine gland consisting of skin and subcutaneous tissue, breast parenchyma (ducts and lobules), and a supporting matrix that includes fat and lymphatic vessels inserted in a complex network of ligaments, including nerves, arteries, and veins [20].

**Fig. 1:**
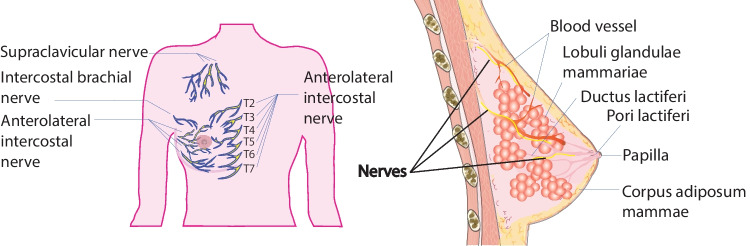
Nerve system in normal breast tissues. Anatomy of the breast and the nerve fibers inside. T2–7 indicate the 2^nd^ to 7^th^ thoracic nerves; yellow, nerve fibers; red, blood vessels

Breasts are innervated by sympathetic and sensory fibers from the 4^th^–6^th^ thoracic nerves [21]. Scientists have examined the mammary gland innervation in rabbits via anatomical, electrophysiological, and histochemical staining of catecholamine and cholinesterase, which mainly reflect sympathetic adrenergic fibers. Sympathetic nerves were observed to innervate the papillary smooth muscle and were present between the media and adventitia of all arteries, without supplying any glandular tissue. In addition, butyrylcholinesterase (bChE)-containing nerve fibers, but not acetylcholinesterase (AChE)-containing nerve fibers, also innervate blood vessels and papillae. The distribution of adrenergic fibers in the papilla and blood vessels is closely correlated with the distribution of bChE-containing fibers, suggesting that a large proportion of the latter is sympathetically derived. Most mammary nerves follow arteries and arterioles. In addition, some sensory nerve fibers leave the arterial network and are located near the walls of the mammary ducts to sense milk pressure (Fig. 1). Currently, there is no evidence of a neural supply for secretory cells or myoepithelial cells or of an innervated sphincter at the duct orifice [22]. In contrast, numerous adrenergic and sensory nerves exist surrounding mammary glands.

### Different roles of sympathetic, parasympathetic, and sensory nerves in breast cancer

Sympathetic and parasympathetic innervations have distinct roles in breast cancer. Sympathetic nerves promote breast cancer progression. The parasympathetic and vagus nerves have tumor-suppressive effects on breast cancer. In a human breast cancer mouse xenograft model and a carcinogen-induced mammary tumor model, breast cancer growth was accelerated after stimulation of sympathetic nerves in the tumor but decreased after stimulation of parasympathetic nerves [16]. Consistently, a retrospective analysis of breast cancer specimens from 29 patients showed that increased sympathetic nerve density and decreased parasympathetic nerve density in tumors were associated with poor clinical outcomes [23]. These neural functions are attribute to β-adrenergic or muscarinic receptors (one type of cholinergic receptor, Chrm1), as well as the tumor microenvironment, and include angiogenesis, tumor-associated macrophages, and adaptive antitumor immunoresponses [24]. α-Adrenergic or β-adrenergic receptor blockers showed tumor-specific denervation, inhibited tumor growth, and downregulated immune checkpoint molecules (programmed death 1 (PD-1), programmed death ligand 1 (PD-L1), and FOXP3), while tumor parasympathetic innervation decreased PD-1 and PD-L1 expression [16].

Additionally, sensory nerves are involved in the tumorigenesis and metastasis of breast cancer. When the brain metastatic breast cancer cells (4TBM) were injected into the mammary pads of BALB/c mice, olvanil, a TRPV1 agonist, activated sensory nerves and significantly inhibited lung and liver metastasis. This process was mainly achieved through enhancement of CD8 + T cell recruitment and increasing IFN-γ responses to irradiated cancer cells by effectively consuming sensory neuropeptides [25]. However, some studies have reported that sensory nerves promote tumorigenesis and metastasis. Co-injection of sensory neurons isolated from the dorsal root ganglia (DRG) of adult female mice with human triple-negative breast cancer (TNBC) cells increased the incidence of lung metastases in immunocompromised mice [26]. These contradictory findings may be explained by the systemic sensory fiber activation due to postnerve injury or the local heterogeneity of sensory fibers. However, when the aggressiveness and inflammatory response increase, the protective role of sensory nerve fibers becomes apparent due to the neuroimmune response. Hence, sympathetic, parasympathetic, and sensory nerves play different roles in breast tumor progression (Table 1).

**Table 1:** The role of sympathetic, parasympathetic, and sensory nerves in breast cancer

Nerve types	Outcome	Clinical application	References
Sympathetic nerve	High nerve density was associated with poor clinical outcomes	β-Blockers or denervation	[15, 20]
Parasympathetic nerve	Low nerve density was associated with poor clinical outcomes	Carbamoylcholine chloride (carbachol), a nonselective Chrm agonist	[15, 20]
Sensory nerves	Activated sensory nerves inhibited lung and liver metastases in mice. Co-culture of DRG-derived sensory neurons of mice with human TNBC cells increased lung metastases in mice	The sensory denervation efficacy of capsaicin injection	[18, 23]

### Innervation differs in breast cancer subtypes

The expression of adrenergic receptors and gamma-aminobutyric acid (GABA) receptors is different in breast cancer subtypes. β-Adrenergic receptor 2 is highly expressed in the luminal A and human epidermal growth factor receptor 2 (HER2) + /estrogen receptor (ER) − subtypes of breast cancer [27, 28]. β-Blockers were found to be associated with a favorable prognosis and improved trastuzumab benefit in patients with HER2-positive early-stage breast cancer [29]. Consistently, β-blockers, such as propranolol, inhibited breast cancer growth and enhanced HER2 therapy sensitivity in preclinical mouse models [27, 30].

Moreover, nerve genes are usually upregulated in TNBC compared with levels in non-TNBC according to The Cancer Genome Atlas (TCGA) database [31]. The pi subunit of the GABA-A receptor (GABRP), GABA transporters, and glutamate decarboxylase (GAD) expression were detected in the normal mammary gland [32]. GAD is the enzyme responsible for catalyzing the synthesis of GABA. GABRP was reported to be highly expressed in the basal-like breast cancer (BLBC) subtype and correlated with poorer outcomes [33], especially breast cancer brain metastasis (BCBM). Depletion of GABRP reduced tumorigenic potential and migration *in vitro*. Mechanistically, GABRP silencing decreased the phosphorylation of extracellular signal–regulated kinase 1/2 (ERK1/2) [33]. Furthermore, GABA enhances the function of aldehyde dehydrogenase (ALDH)1A3, a cancer stem cell (CSC) marker in TNBC [34]. In mice harboring MDA-MB-231 tumors overexpressing ALDH1A3, GABA treatment increased breast cancer metastasis [34]. In addition, breast cancer patient datasets from TCGA, Molecular Taxonomy of Breast Cancer International Consortium (METABRIC), and the Gene Expression Omnibus (GEO) support the co-expression of ALDH1A3 and GABA pathway genes and the corresponding increased risk of metastasis [34].

## Interaction between nerves and breast cancer cells

### Innervation promotes breast cancer progression

**Fig. 2:**
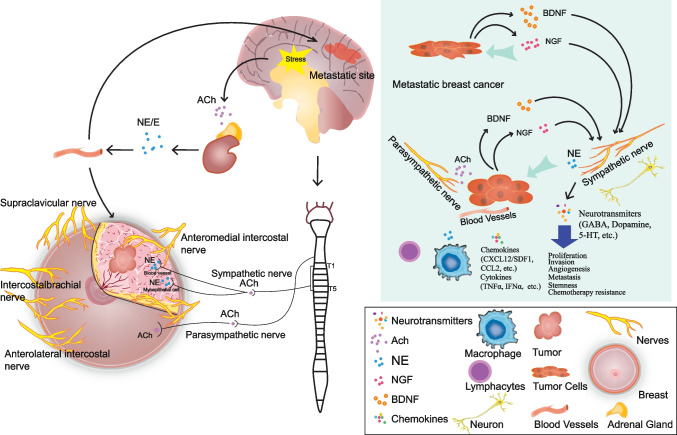
Interactions among breast cancer, innervation, and brain metastasis. Workflow of how stress or other stimulations interact with the peripheral nervous system (sympathetic and parasympathetic nerves), including through the HPA axis, leading to the release of several neurotransmitters and hormones and promoting breast cancer growth and brain metastasis. In turn, the tumor cells in the metastatic site promote tumor growth directly or indirectly through neurons

The neuro-malignancy-promoting circuit is initiated by cancer cells that release axonal neurotrophic axonal factors, which directly link the tumor stroma to the peripheral nervous system (PNS) (Fig. 2). In return, nerve endings in the tumor microenvironment (which can be of adrenergic, cholinergic, or sensory original nerves) release neurotransmitters that stimulate corresponding receptors in stromal cells, immune cells, and cancer cells, thereby modulating cancer growth and metastasis. In brain metastatic breast cancer, gap junctions are formed between breast cancer cells and astrocytes, transferring cGAMPs from cancer cells to astrocytes and producing cytokines, such as tumor necrosis factor alpha (TNF-α) and interferon alpha (IFN-α). These factors further stimulate the NF-κB and STAT1 signaling pathways in brain metastatic cells, thereby promoting cancer growth and chemotherapy resistance [35, 36].

Metastasis is one of the hallmarks of cancer. In a clinical study, nerve infiltration was observed in 28% of lymph node–positive patients, demonstrating a potential role of innervation in breast tumor metastasis [8]. In several preclinical mouse studies, neural infiltration was reported to promote breast cancer bone metastasis [37, 38]. Additionally, epinephrine (E) and norepinephrine (NE) are involved in breast cancer metastasis through α- or β-adrenergic receptors. Adrenergic signaling–driven innervation promotes breast cancer metastasis by accelerating bone degradation and bone vasculature and remodeling processes. Native bone vasculature and remodeling processes are involved in driving breast cancer cell engraftment in bone [39].

### Innervation promotes breast cancer brain metastasis

Most breast cancer patients, especially those with an aggressive basal-like subtype, die from metastasis to the lung, bone, liver, and especially, the brain [40]. Breast cancer is one of the top five primary tumors that develop brain metastases. Brain metastasis has a 5-year cumulative incidence in 7% of breast cancer patients [41]. Brain metastasis patients have only a 20% probability of surviving more than a year [42]. Most brain metastatic sites occur in the parenchyma area, mainly due to the hematogenous spread, while rare metastases in the leptomeningeal and vertebral areas mostly spread along nerves or perineural lymphatics [43].

The successful colonization of breast cancer cells in the brain is quite challenging due to the harsh environment in the brain. The following steps are required for the habitation and colonization:

1. Crossing the blood–brain barrier (BBB): Exosomes from highly metastatic cells increase BBB permeability *in vitro* and in BBB models [44].
2. Dormancy after colonization: Most disseminated tumor cells undergo a variable period of dormancy before relapsing in the brain [45].
3. Habitation of tumor cell behavior and nutrition: Synapses of tumor cells have been observed at metastatic sites [46].

Specifically, breast cancer cells in the brain microenvironment express GABA-related genes, enabling them to utilize GABA as a metabolite for a proliferative advantage [47]. Since metastatic cells cannot obtain enough glutamate in the brain environment, metastatic cancer cells have been found to activate the *N*-methyl-d-aspartate receptor (NMDAR), forming a pseudotriad synapse between cancer cells and glutamatergic neurons [48]. The activation of NMDAR-mediated colonization and the reprogrammed metastatic-innervated niche contribute to cancer cell survival [14]. In addition, the brain metastasis mechanism was reported to be mediated by astrocyte-derived miR-19a through inhibition of phosphatase and tensin homolog (PTEN) expression in breast cancer and melanoma, thereby promoting brain metastasis. When passing through the BBB, the metastatic tumor cells successfully reverse the brain metastatic microenvironment to facilitate the growth of tumor cells after a long dormant period [49].

#### HER2 amplification promotes brain metastasis

*HER2* gene amplification is an important risk factor for brain metastasis. The *HER2* gene encodes a transmembrane receptor tyrosine kinase that is elevated in 20–30% of human breast cancers [50]. In approximately 40% of patients with HER2 + breast cancer or TNBC, the cancer cells metastasize to the brain [51]. The association between HER2 overexpression in primary breast cancer and subsequent brain metastasis is 97% [52]. Approximately 90% of HER2 + patients will die from brain metastases [42]. In detail, the invasiveness of HER2 + breast cancer was reported to be associated with upregulated CXCR4, increasing the permeability of brain endothelial cells and promoting breast cancer cell invasion. Although HER2-directed therapy can target HER2 + breast cancer in the metastatic site [53], HER2-targeted therapies failed to reduce brain metastasis in several studies [54, 55]. Lapatinib is an irreversible tyrosine kinase inhibitor targeting HER2 with the ability to penetrate the BBB [47]. In addition, pyrotinib is an alternative strategy for the treatment of brain metastatic breast cancer, especially HER2-positive breast cancer, and has been verified to improve overall survival when combined with surgery and radiotherapy [56].

#### Immune regulation involved in brain metastasis

The brain metastasis oncogenic lncRNA (*BMOR*) gene promotes specific distal brain metastasis. Mechanistic studies have been revealed that cancer cells are prevented from being killed by immunity in the brain microenvironment through the BMOR-IFR3 pathway [57]. Central nervous system (CNS) myeloid cells, namely microglia and border-associated macrophages, are the major immune cell population in brain homeostasis and contribute significantly to brain disease. CNS myeloid cells promote BCBM by downregulating the expression of CX3CR1, which in turn upregulates the expression of the chemokine ligand CXCL10. Remarkably, neutralization of CXCL10 was reported to decrease BCBM, whereas CXCL10 overexpression increased BCBM by recruiting VISTA^Hi^ PD-L1^+^ CNS marrow to metastatic lesions. Inhibition of VISTA and PD-L1 signaling has been shown to alleviate immunosuppression and reduce BCBM [58].

In BCBM, PD-L1 and PD-L2 expression is approximately 53% and 36%, respectively [59]. In several clinical studies, PD-L1 expression was found to be significantly correlated with better survival. The survival relationship between PD-L1 expression in breast cancer and better outcomes can be explained by the presence of a strong antitumor immune response, including a high number of CD8 + T cells [60] and IFN-γ release, leading to PD-L1 upregulation [61]. Therefore, PD-L1 expression on tumor-infiltrating lymphocytes is a beneficial biomarker in prognosis and antitumor immunity, indicating the effectiveness of immune checkpoint inhibitors in BCBM [59]. Consistently, in operable breast cancer cases, a high CD8 + /PD-1 + ratio was significantly correlated with long overall survival, although tumor-infiltrating PD-1 + levels were associated with shorter overall survival [62]. Moreover, strong expression of PD-L1 and PD-L2 in the cell membrane of breast cancer cells mainly results from the alteration of several signaling pathways, including PTEN, phosphoinositide 3-kinase (PI3K), and mechanistic target of rapamycin (mTOR) [63]. PTEN loss and PI3K activation were reported to upregulate PD-L1 in the breast cancer cell membrane [64].

The BBB is often compromised with the development of brain metastases, allowing multiple immunosuppressive cells to infiltrate from periphery circulation [65]. As a type of brain cell, astrocytes regulate the brain response to inflammation [66]. In recent years, astrocytes have been reported to drive the spread of cancer to the brain by secreting interleukins (ILs) and chemokines, such as CCL2 and CXCL12/SDF1 [67], as well as by activating PPARγ, which in turn helps cancer cells gain a foothold in the brain [68, 69]. In addition, cancer cells that metastasize to the brain can interact with astrocytes by releasing different cytokines and inflammatory mediators [70], including IL-6, transforming growth factor beta (TGF-β), insulin-like growth factor 1 (IGF-1), and CXCL12α. Meanwhile, brain metastatic breast cancer can recruit type 2 tumor-associated macrophages [71–74], which play a negative role in antitumor immune responses by reducing the key molecules (including CD80, CD86, CD40, and so on) involved in T cell activation [75].

### Nerves drive tumor angiogenesis and lymphangiogenesis

Tumor angiogenesis is one of the major prerequisites for tumor growth, which relies on an adequate supply of oxygen and nutrients. Likewise, new lymphatic vessels are developed in tumors to facilitate tumor cell metastasis [76]. Neuronal markers observed in tumor tissue are prognostic cancer markers [77]. Neurotransmitters and growth factors released from nerve terminals not only stimulate the proliferation of cancer cells but also promote angiogenesis [23]. In breast cancer cell lines, direct activation of β-adrenergic signaling amplifies the expression of vascular endothelial growth factor (VEGF), IL-6, and IL-8, which stimulate angiogenesis [78]. Nerve growth factor (NGF) and brain-derived neurotrophic factor (BDNF) released from breast cancer cells were reported to induce the growth of nerve fibers along breast ducts, blood vessels, and lymphatic vessels in breast tumor tissues. Notably, neurotransmitters released from adrenergic nerves stimulate angiogenesis, which promotes breast tumor growth and metastasis [79].

### Innervation modulates the immune response in breast cancer

#### Adrenergic nerves promote breast cancer progression through immune suppression

Crosstalk between neural and immune cells is important in cancer immunity and inflammation [80]. Adrenergic signaling is necessary for the generation, infiltration, and activation of immune cells in the tumor microenvironment (TME), resulting in lung and lymph node metastasis [81]. In addition, stress-related catecholamines regulate lung colonization by recruiting immune cells. β-Adrenergic signaling increases the infiltration of CD11b + F4/80 + macrophages into the primary tumor parenchyma, which upregulates prometastatic gene expression and M2 macrophage infiltration [81]. In addition, β-adrenergic signaling promotes lung metastatic colonization by increasing monocyte export and macrophage infiltration. Pharmacological inactivation of β-adrenergic signaling with propranolol reversed stress-induced macrophage infiltration and inhibited tumor progression to distant tissue spread in mice [81].

Activation of the sympathetic nervous system (SNS), release of neurotransmitters, and adrenergic receptor (AR) signaling regulate solid tumor growth and metastasis [18]. A retrospective analysis of 29 breast cancer patients showed that increased sympathetic nerve density and decreased parasympathetic nerve density in tumors were associated with higher expression of immune checkpoint molecules and poor clinical outcomes [16]. Specific inhibition of the sympathetic nerve significantly inhibited tumor growth and downregulated immune checkpoint molecules (PD-1, PD-L1, and FOXP3), which was far more effective than pharmacological blockade of the α- or β-adrenergic receptor. All these findings suggest that autonomic innervation of tumors regulates breast cancer through the immune system.

#### Vagal nerves inhibit breast cancer through anti-inflammatory activity

Vagal nerves play key roles in regulating the visceral homeostasis and are beneficial for tumor development through the anti-inflammatory hypothalamic–pituitary–adrenal (HPA) axis [82]. Several clinical trials (NCT01569503, NCT02311660, NCT01552941, NCT01572155, NCT02425774) found that the vagal nerve stimulation therapy reduced chronic inflammation [82] due to its anti-inflammatory properties through acetylcholine (ACh) and the α-7-nicotinic ACh receptor (α7nAChR), since α7nAChRs are widely expressed in a variety of immune cells, including lymphocytes, macrophages, mast cells, dendritic cells, and lymphoid and myeloid cells [83]. ACh binds to α7nAChR in macrophages to inhibit the release of TNF-α. Additionally, substance P (SP) is a neuropeptide found in vagal afferent (sensory) fibers [84] and was found to inhibit cancer initiation and progression and modulate immune responses.

Vagal nerve activity and inactivation of sensory neurons have been shown to enhance visceral metastasis in 4THM breast cancer. In a 4THM breast cancer cell mouse model, vagotomy or semapimod (CNI-1493) can activate the vagal nerve. Semapimod treatment reduced lung and liver metastasis compared with vagotomized animals, and the SP levels were increased in sensory nerve endings as well [85]. Consistently, breast cancer metastasis can be promoted by surgical removal of vagal activity (vagotomy). Systemic inhibition of sensory nerve activity by high-dose capsaicin increases lung and cardiac breast cancer metastasis [86]. Therefore, vagal nerve activity is important in controlling the visceral metastasis of breast cancer.

## Cancer cells trigger innervation through neurotransmitters and intrinsic neural expression

The transmission of information between neurons and effector cells, such as muscle cells and gland cells, occurs mainly through neurotransmitters. Neurotransmitters can be divided into cholines (ACh [87]), monoamines (catecholamines, including NE, E [88], dopamine [16], and 5-hydroxytryptamine (5-HT) [89], and histamine [90]), amino acids (excitatory transmitters, such as glutamate [91] and aspartic acid [92]; inhibitory transmitters, such as GABA [93], glycine [94], and taurine [95]), polypeptides (neuropeptides [96]), purines [97] (adenosine, adenosine triphosphate), and gaseous substances (such as nitric oxide [98]). When a neuron is stimulated by signals from the environment or other neurons, the transmitters stored in the presynaptic vesicles can be released to the synaptic cleft and act on the postsynaptic membrane, a process which is quite fast and ends when transmitters are digested or degraded by corresponding hydrolases or re-take up by presynaptic membrane-specific transmitter transporters [48]. Tumor cells were found to be able to synthesize and release a variety of neurotransmitters to induce different biological effects through their receptors [14, 96] (Table 2). Therefore, through the transmitters released and intrinsic neural genes and receptors expressed, cancer progression drives axonogenesis and neurogenesis, stimulates neural reprogramming, and has a crosstalk among the tumor, microenvironment, and nerves [99].

**Table 2:** Neurotransmitters and their receptors in breast cancer

Properties	Molecule	Receptor	Function	References
Cholines	Acetylcholine (ACh)	Adrenergic receptor (α1D2, β1, β2, β2/3)	α2-AR increases tumor growth and metastases, and β1-AR promotes tumor growth and metastasis and causes infiltration of macrophages into tumor lesions; β2-AR inhibits cell proliferation, angiogenesis, and immune response	[26, 83, 103, 104]
Monoamines	Catecholamines	β-ARs	Promotes breast cancer cell migration and metastasis, is associated with poor prognosis, and causes infiltration of macrophages into tumor lesions, promoting metastasis	[77, 95, 97]
	Norepinephrine (NE), epinephrine		Promote tumor cell proliferation, survival, and migration directly	[36, 95, 98]
	Dopamine	D1, D2, D3, D4, and D5 receptors	D2R induces cancer stem cell survival; dopamine antagonists are associated breast cancer risk Dopamine inhibits T cell proliferation and cytotoxic capacity via D1 receptors	[110, 111, 117]
	Serotonin (5-HT)	5-HT R	5-HT promotes proliferation and invasion through the 5-HT7 receptor and increases TPH1 and VEGF expression	[85, 122, 123]
	Histamine	H1	H1 receptor inhibition enhances antitumor responses through ERK activation	[86]
Amino acids	Glutamate (Glu)	mGluR1/3, NMDAR, AMPA receptor	Promote cell proliferation, invasion, and metastasis	[29, 87, 153–155]
	Glycine		Promotes brain metastasis by activating NMDAR	[90, 155]
Inhibitory transmitters	Gamma-aminobutyric acid	GABAA and GABAC receptors	Promote tumor cell proliferation and progression through cytokeratins and phosphorylation of ERK1/2 and promote metastasis by ALDH1A3 and immune response	[29–31]
	Taurine		Results in a more differentiated phenotype	[92]
Polypeptides	Neuropeptides			[92]
	Substance P (SP)	NK1 receptor induces	Transactivates EGFR and HER in cancer cells, exerts anticancer functions, but also promotes proliferation and invasion	[81, 204]
Purines	Adenosine triphosphate (ATP)		Be converted into cAMP to activate protein kinase A	[96, 102]
Gaseous substances	Nitric oxide (NO)		Promotes progression by activating ERK1/2, PI3K/AKT, and c-Myc	[94]
	Brain-derived neurotrophic factors (BDNF)	Tropomyosin-related kinase B (TRKB; also known as NTRK2)	Promotes angiogenesis, proliferation, migration, and invasion	[172, 178, 181, 182]
Neurotrophins	Nerve growth factor (NGF)	TRKA, p75NTR	Promotes tumor aggressiveness and tumor growth and inhibits p53 activity	[172, 173, 209]
	Neurotrophins (NT)	Cholinergic muscarinic receptor (Chrm1)	Stimulates breast cancer cell survival, enhances breast cancer brain metastasis, and contributes to breast cancer cell survival	[21, 172]
	Netrin-1	Deleted in colorectal cancer (DCC) and uncoordinated 5 homologs (UNC5H)	Promotes tumorigenesis and metastasis, as a diagnostic marker	[194, 197]

### Catecholamines and β-adrenergic receptors

Catecholamines are monoamine neurotransmitters synthesized and secreted by the adrenal medulla, adrenal neurons, and extra-adrenal chromaffin bodies. Catecholamines include dopamine, E, and NE. E and NE have been reported to directly promote tumor cell proliferation, survival, and migration. Increased E and NE caused by psychological stress and depression favor cancer development [100]. Adrenergic receptors include α receptors and β receptors; the former cause vasoconstriction and mydriasis, while the latter cause cardiac excitation, vascular changes and bronchiectasis, and metabolic changes. The α1 receptor, located on the cell membrane (postsynaptic membrane) of noradrenergic innervated effector organs, is agonized by phenylephrine and methoxyamine and blocked by piprazole. α2 Receptors are activated by clonidine and blocked by yohimbine and are located on the presynaptic membrane, the membranes of adipocytes, and the membranes of some visceral and vascular smooth muscle cells. β Receptors, including β1, β2, and β3, bind to G proteins and activate adenylate cyclase to convert adenosine triphosphate (ATP) into cyclic adenosine monophosphate (cAMP), which in turn activates protein kinase A (PKA) [101].

The tumor-promoting activities of catecholamines predominantly depend on the activation of β-ARs. Increased expression of β-ARs for catecholamines is associated with poor prognosis in breast cancer [102]. β-AR activation promotes breast cancer cell migration and metastasis [81] and angiogenesis via the secretion of VEGF and IL-6 in various cancer cell lines [103]. The β-adrenergic signaling pathway activated by stress caused the infiltration of macrophages into tumor lesions, promoting metastasis in an orthotopic breast cancer model in BALB/c mice [81]. β-Blocker treatment suppresses metastasis and disease recurrence and improves breast cancer patient survival [104]. Moreover, β-blockers have a preventive effect on cancer development in different models [105]. Several population-based studies suggest that patients taking adrenergic antagonists for cardiovascular disease may have a significantly lower incidence of breast cancer [106]. The β1-blocker nebivolol specifically blocks oxidative phosphorylation in cancer cells by synergistically inhibiting complex I and ATP synthase activity [107]. Endothelial cells express β1-AR, and nebivolol significantly reduces glycolytic flux in endothelial cells [108] and further prevents endothelial cell proliferation and tumor angiogenesis [107].

However, there is an opposing view on the function of β2-AR in breast cancer prognosis. Positive β2-AR expression, being associated with inhibition of cell proliferation, angiogenesis, and the immune response, showed a good prognosis value in HER2-positive breast cancer patients [29]. In the 4T1 model, activation of α2-ARs increased tumor growth and metastases [109]. In contrast, blocking α-ARs in the absence of stress increased distant metastasis in an orthotopic mouse model of breast cancer [110].

### ACh and cholinergic receptors

The parasympathetic system plays an important role in tumor innervation apart from the sympathetic systems. ACh is a neurotransmitter of the parasympathetic nervous system, and its biological activity is mediated by a nicotinic ACh receptor (nAChR) and muscarinic ACh receptor (mAChR) in the central nervous system and PNSs. Nicotine binds to nAChRs to promote cancer cell proliferation and invasion, including breast cancer cells. NAChRs can also regulate the synthesis and release of catecholamines in the adrenal medulla and sympathetic nerve endings. Therefore, combining cigarette smoke exposure with stress stimulation can accelerate tumor growth more than smoking or stress alone [111]. Nicotine pretreatment can stimulate the activation of α9-nAChR, promoting MCF-7 and MDA-MB-231 breast cancer cell migration [112]. MAChRs are another family of ACh receptors that activate MAPK to induce cell proliferation and protein synthesis in human breast cancer cells [113].

The vagus nerve is an important part of the parasympathetic system. The vagus nerve mainly inhibits tumorigenesis directly through a cholinergic mechanism in breast cancer. When adeno-associated virus (AAV) was injected into both human breast xenograft and transgenic mouse models to express long-acting bacterial sodium channels in tumor cholinergic nerves, the breast tumor growth was reduced [16]. Consistently, cholinergic stimulation also inhibits breast cancer by reducing PD-1 expression levels in CD4 + and CD8 + lymphocytes [114].

### Dopamine and dopaminergic receptors

Dopamine is an extremely important neurotransmitter in the brain, accounting for approximately 80% of the catecholamine content in the brain. Five dopamine receptors have been identified and are divided into D1-type receptors (including D1 and D5) and D2-type receptors (D2, D3, and D4). Dopamine receptors are G protein–coupled receptors (GPCRs). Elevated D2 expression was detected in breast cancer [115]. According to the TCGA database, D1, D2, and D5 are highly expressed in adjacent normal tissues but expressed at low levels in breast cancer, while D4 is highly expressed in breast cancer but expressed at low levels in adjacent tissues. There is no such difference in D3 between breast cancer and adjacent tissue [116].

Moreover, dopamine or dopamine receptor agonists seem to exert inhibitory effects on breast cancer growth [117, 118]. Dopamine antagonists were found to be associated with an increased risk of cancer, with a dose–response relationship between a larger cumulative dose and a higher risk. Consistently, the use of antipsychotic dopamine antagonists may be associated with a small but significant risk of breast cancer [119]. The combination of dopamine or dopamine receptor agonists with anticancer drugs (such as doxorubicin or 5-fluorouracil, 5-FU) [120] can inhibit the growth of various cancer types, including breast cancer. Nonetheless, dopamine itself does not affect cancer growth. The major anticancer effects are related to the inhibition of tumor endothelial cell proliferation and migration by inhibiting VEGF receptor 2 phosphorylation and MAPK activation. These effects are mainly mediated by the D2 receptor expressed in tumor endothelial cells [121, 122].

However, dopaminergic receptors (DRs) were also observed to be highly expressed in CSCs [123] and can be targeted by DR antagonists to inhibit the proliferation of CSCs. Normal human pluripotent stem cells do not express DRs, while CSCs express all five DRs. CD44 + CD24 − CSCs express DRs in TNBC [123]. The DR antagonist thioridazine acts on the D2 receptor expressed on CSCs, specifically inhibiting CSC proliferation but not affecting normal human stem cells [124]. The D2R antagonists trifluoperazine and thioridazine eradicate CSCs in breast cancers resistant to conventional therapy [122]. Mechanistically, D2R antagonists reduce CSC proliferation and invasion by inhibiting the ERK and AKT pathways and by downregulating the expression of matrix metalloproteinase (MMP)-9 and octamer-binding transcription factor 4 (Oct-4) [125]. Additionally, D4 has also been found to be overexpressed in breast cancer, while D5 was in a low level of both breast cancer and normal breast tissues. D5 was reported to promote autophagy by increasing ROS production and inhibiting the mTOR pathway in a subset of breast cancers [126].

### Serotonin and serotonin receptors

Serotonin, also known as 5-HT, is an inhibitory neurotransmitter with high content in the cerebral cortex and synapses. Serotonin receptors are located in the cell membranes and mediate the functions of serotonin and several neurotransmitters. Thus, serotonin receptors affect a variety of biological and neurological processes and are targets of a wide variety of drugs, including antidepressants and antipsychotics. Except for the 5-HT3 receptor, which is a ligand-gated ion channel, all other serotonin receptors are GPCRs that activate intracellular second messenger cascades [127, 128].

5-HT is a key local regulator of epithelial homeostasis in the breast. Human breast cancer induces complex changes in the mammary serotonin system. In normal mammary glands, 5-HT acts as a physiological regulator of lactation and involution, promoting growth arrest and cell death. However, in breast cancer cells, especially in TNBC cells, tryptophan hydroxylase (TPH)1 expression is increased, causing resistance to 5-HT-induced apoptosis [129]. For example, in MDA-MB-231 cells, 5-HT increased TPH1 and VEGF protein expression and promoted proliferation and invasion through the 5-HT7 receptor. In addition, 5-HT was found to have a stronger effect in the early stage of metastasis than in the later stage [89], showing an important role in breast cancer progression.

In addition, enzymes, such as monoamine oxidase A (MAOA) [130] that degrade catecholamines and serotonin, play an important role in cancer metastasis. Several studies have reported that a low level of MAOA is correlated with breast cancer invasion and that the inhibition of MAOA stimulates the malignant behavior of MDA-MB-231 cells [131, 132]. In contrast, MAOA was also reported to promote tumor sphere formation and might drive breast tumor–initiating cells [133]. While serotonin reuptake transporter inhibitors and serotonin receptor inhibitors were found to inhibit mouse breast cancer–initiating cell activity, serotonin transporter antagonists were found to target tumor-initiating cells in a transgenic mouse model of breast cancer [134]. The selective serotonin reuptake inhibitor (SSRI) sertraline (Zoloft) was reported to promote the efficiency of docetaxel in controlling breast tumor growth [134]. In addition, studies of 6959 consecutive newly diagnosed breast cancer patients in northern Israel showed that selective SSRI use within 5 years prior to breast cancer diagnosis was associated with a 66% increase in overall mortality, while after diagnosis, the use of SSRIs was associated with an 81% increase in mortality [135].

Depression is common in breast cancer patients, approximately 32.8% have depressive symptoms, and 42% of recurrent cases were diagnosed with depression [136]. A recent study reported the antitumor potential of 5-HT receptor 1A (HTR1A) in breast cancer cells [137]. HTR1A interacts with TRIM21 to inhibit the TGF-β pathway [137]. Thus, HTR1A is an independent predictive factor for breast cancer patients and might provide a new approach for TNBC treatment. Therefore, 5-HT, its receptors, and the related enzyme MAOA can play both promotive and inhibitory roles in breast cancer progression. However, the underlying mechanisms behind are still unclear.

### GABA and GABAergic receptors

GABA is a major inhibitory neurotransmitter synthesized from glutamine, glutamate, and glucose by GAD65 and GAD67. GABA binds to GABA-A, GABA-B, and GABA-C receptors with different functions. GAD is only expressed in normal nervous tissues; however, recent studies have shown that GABA can also be secreted by cancer cells, dendritic cells, T cells, natural killer (NK) cells, macrophages, and B cells [138].

GABA levels are positively correlated with breast cancer metastasis. The expression of GABA-A receptors, GABA transporters, and GABA transaminases was found to be significantly upregulated in brain metastatic breast cancer cells compared with primary breast cancer cell lines. GAD and GABRP were also upregulated [139]. Almost 50% of all breast cancer types exhibit high GABRP levels [139], which are usually elevated both in circulating breast cancer cells and in metastatic lymph nodes isolated from breast cancer patients [140]. Patients with metastatic breast cancer express eightfold higher GABRP levels than stage II–IV patients without evidence of metastasis [141] and 30-fold higher levels than stage I patients without evidence of metastasis, suggesting that the GABRP expression level increases with metastasis. Elevated GABRP expression levels were also effective in detecting circulating tumor cells in stage I (65%), stages II–IV without evidence of metastasis (72%), and metastatic (88.5%) breast cancer patients. Since circulating tumor cells are an important indicator of overall survival in breast cancer patients [142], detection of GABRP could be used to monitor disease progression.

GABRP could be a promising marker and therapeutic target for BLBC [33]. Normal mammary luminal progenitor cells, which give rise to BLBC cells during carcinogenesis, express high levels of GABRP [143], further suggesting a strong link between GABRP and BLBC subtypes [144, 145]. GABRP knockdown inhibited TNBC cell proliferation *in vitro* and inhibited the growth of MDA-MB-468 xenografts in nude mice [33]. Knockdown of GABRP in BLBC cell lines decreased the phosphorylation of ERK1/2 [33]. Similarly, antitumor properties were observed when knocking down GABRP gene expression or employing a GABRP antibody [146], further underscoring the prospective role of GABRP as a therapeutic target in breast cancer.

Elevated expression of GABA and GABA-A receptors reshapes brain metastatic breast cancer cells to behave like neurons. BLBC patients often develop distant metastasis in the lung, liver, and brain [147, 148]. Breast-to-brain metastatic cells display a GABAergic phenotype similar to that of neuronal cells. GABA-A receptors, GABA transporters, GABA transaminases, parvalbumin, and reelin are all highly expressed in brain metastatic breast cancer cells so that these cancer cells can take up GABA and catabolize it to succinate, which serves as a biosynthetic source, thereby gaining a proliferation advantage [32]. Nevertheless, metastatic breast cancer cells mimic neuron-like cells, allowing breast cancer cells to adapt to the brain microenvironment and use GABA as a metabolite [149].

GABA promotes breast cancer metastasis through ALDH1A3. High ALDH activity (due to ALDH1A3 isoform activity) is a well-known feature of breast CSCs and is associated with a poorer prognosis, including tumor growth, invasion, metastasis, and chemoresistance [150, 151]. High expression of both ALDH1A3 and GABA resulted in increased lung and brain metastasis in mice bearing orthotopic MDA-MB-231 tumors [33]. Metabolite analysis of patient tumor samples demonstrated that GABA promotes breast cancer metastases through GABA metabolism, which is associated with ALDH1A3. High levels of ALDH1A3 in breast tumor cells convert GABA into *N*-acetylputrescine and glutamate [34]. *N*-Acetylputrescine is a polyamine metabolized by amino acids such as l-ornithine, lysine, and l-arginine. An increasing number of reports in the literature have now identified polyamine catabolism as a contributory factor in the development of many cancer types, including breast cancer [152, 153].

Additionally, GABA promotes breast tumor progression by regulating the immune response. Abundant glutamine is available in the tumor environment to synthesize GABA [154]. Blockage of GABA can activate the expression of specific cytokines (such as Ccl4 and Ccl5) in the tumor, which promotes the infiltration of dendritic cells and T cells. When B cells are activated, they release GABA to promote monocyte differentiation into anti-inflammatory macrophages and suppress antitumor CD8 + T cell responses through IL-10. GABA can bind to and activate GABA type A receptors (GABA-A-Rs) on nearby immune cells, such as macrophages and CD8 + T cells. In breast cancer, the GABA-B receptor was reported to interact with CXCR4, a receptor for the chemokine CXCL12 (stromal cell–derived factor 1α). Furthermore, both GABAB antagonists and agonists can block CXCL12-elicited chemotaxis, possibly by influencing high-threshold Ca^2+^ channels [155].

### Glutamate and NMDARs

Glutamate, a nonessential amino acid, is both a major bioenergetic substrate in cells and an excitatory neurotransmitter involved in biosynthesis, bioenergetics, metabolism, and oncogenic signaling [156]. Interestingly, there is growing evidence that glutamate can function through its receptors in human malignancies. Dysregulation of glutamate transporters, such as excitatory amino acid transporters and the cystine/glutamate antitransport system, in turn activates glutamate receptors on cancer cells and leads to malignant growth [157]. According to a metabolic analysis of 270 clinical breast cancer samples and 97 normal breast tissues, 56% of ER + tumor tissues and 88% of ER − tumor tissues are glutamate rich [158]. A higher glutamate-to-glutamine ratio was significantly correlated with ER status, a higher tumor grade, and prolonged overall survival. A possible explanation is that patients with glutamate-enriched tumors could be more sensitive to cytotoxic chemotherapy. And, this is a retrospective cohort analysis with a limited median follow-up time of 40 months in 249 patients (28 deaths). ER-negative and selective ER-positive breast cancers are more sensitive to the recently discovered glutaminase inhibitors (BPTES and 968) [158].

Moreover, breast cancer cells secrete high levels of glutamate and frequently metastasize to bone. Exogenous glutamate disrupts normal bone turnover and may contribute to cancer-induced bone pain (CIBP). Scientists have identified effective inhibitors of glutamate secretion in MDA-MB-231 and MCF-7 cells [159]. These inhibitors targeting glutamate release may provide a novel strategy for inhibiting pain propagation by blocking the mechanism causing the pain, especially those caused by metastases.

Neuronal and non-neuronal brain cells express different classes of glutamate receptors, which are divided into ionotropic glutamate receptors (iGluRs) and metabotropic glutamate receptors (mGluRs/Grms) [160]. iGluRs are quaternary ligand-gated ion channels classified into 3 subfamilies: *N*-methyl-d-aspartate (NMDA) receptors, α-amino-3-hydroxy-5-methyl-4-isooxazole propionate (AMPA) receptors, and kainic acid receptors [161]. As glutamate-gated ion channel receptors, NMDARs have been detected in different tumors [162]. NMDARs are activated by binding to glutamate or glycine, resulting in calcium ion entry and membrane depolarization. Activation of NMDARs promotes breast cancer metastasis to the brain [48]. NMDAR subunits are highly expressed in brain metastatic breast cancer cell lines. Knockdown of an NMDAR subunit suppressed tumor growth and increased brain metastasis-free survival in subjects but did not affect their primary tumor burden or lung metastases [99]. Furthermore, NMDAR activation limits the duration of signaling by ERK1/2, which dampens mTOR signaling activity [163]. NMDAR antagonists (MK-801 or dizocilpine) inhibit breast cancer cell proliferation and invasion through the mTOR and ERK signaling pathways [163]. Given the antiproliferative effects of NMDAR agonists, clinical trials and precise patient selection may validate the therapeutic potential of these emerging antineoplastic agents [164].

Glutamate antagonists have shown potential enhancing effects when combined with cytostatic drug therapy in breast cancer [91]. AMPA antagonists (GYKI52466) inhibit breast cancer cell proliferation and invasion. Breast cancer cells were sensitive to the antiproliferative effect of NMDAR antagonists (dizocilpine) and GYKI52466. Glutamate antagonists contribute to antiproliferation through Ca^2+^ channels and altered Ca^2+^ concentrations. Tumor cell morphology can also be changed because of reduced membrane ruffling and pseudopodial protrusions. Therefore, both NMDAR and AMPA antagonists have potential therapeutic effects in breast cancer treatment [91].

mGluR1 is a member of the GPCR superfamily that participates in synaptic transmission and neuronal excitability. Aberrant mGluR1 expression was detected and explored as a novel target for breast cancer treatment in a clinical study. In a microarray containing 394 consecutive primary breast cancer tissues, approximately 56% (*n* = 219) of breast cancer tissues showed mGluR1 expression. mGluR1 expression (31% (*n* = 18/58)) was significantly associated with metastasis-free survival (MFS) in ER-negative tumors. mGluR1 expression (11/44) in TNBC was significantly associated with shorter MFS and with poor OS. In conclusion, mGluR1 is frequently expressed and plays a role as an unfavorable prognostic marker in TNBC [165]. There were 131 genes differentially expressed in mGluR1-silenced MDA-MB-231 cells compared with control cells. Four major canonical pathways associated with acute inflammation (such as upregulation of CXCL1, IL-6, and IL-8) were included. Therefore, mGluR1 was identified as a novel endogenous regulator of inflammation in TNBC [166].

In addition, mGluR1-expressing immature mammary epithelial cells exhibited delayed lumen formation. The implantation of mGluR1-expressing immature mammary epithelial cells into nude mouse mammary fat pads resulted in mammary tumor formation. Consistently, mGluR1 was observed to be expressed in human breast cancer cell lines and breast tumor biopsies. Furthermore, the glutamate release inhibitor and an AKT inhibitor led to tumor growth inhibition in MCF-7 xenografts. Therefore, mGluR1 plays an oncogenic role in breast cancer and could be a potential therapeutic target [167].

### Neprilysin

Neprilysin is a cell surface peptidase that is able to degrade angiotensin II, endothelin I, and substance P, among others. Neprilysin is expressed in various tissues including the breast; however, neprilysin is downregulated in breast tumor tissues. The tumor-suppressor mechanisms of neprilysin mainly rely on its peptidase activity and its protein–protein interaction with PTEN, focal adhesion kinase, ezrin/radixin/moesin, and PI3K. Neprilysin can be downregulated by histone deacetylation and hypermethylation of the neprilysin gene promoter region, resulting in angiogenesis, cell survival, and cell migration [168].

### Endorphins

Endorphins are mood elevators that can be stimulated by physical activity [169]. A home-based physical activity intervention was found to be related to fewer mood disturbances among breast cancer survivors [170]. Similarly, breast cancer survivors were observed to have more positive moods after completing an exercise intervention. In addition, physical activity prevents survivors from experiencing disease stress, provides them opportunities for social interactions, and improves self-confidence and body image. A clinical study used an adaptive cognitive-behavioral stress management intervention to treat 114 women diagnosed with breast cancer (any stage of disease, any type of breast cancer) and found that physical activity interventions may improve quality of life and mood in this population [171].

### Melatonin

Melatonin, an indoleamine that is highly available during darkness, is a hormone derived from tryptophan and secreted by the pineal gland. Melatonin is naturally occurring in mammals and has been shown to have a variety of antitumor effects targeting different molecules and signaling pathways, such as angiogenesis, remodeling of the extracellular matrix, migration, and epithelial-to-mesenchymal transition (EMT) in many cancer types, including breast cancer [172]. Melatonin treatment in the daytime in human breast cancer xenografts regulated plasma metabolites and thus controlled the aggressiveness at an early stage [173].

In addition, melatonin treatment caused the upregulation of Bax expression but the downregulation of Bcl-2 expression, thus inhibiting the growth of breast cancer cells by inducing cell death in both MCF-7 and MDA-MB-231 cells [174]. In addition, in breast cancer, melatonin was reported to play a role in cancer-inhibitory processes through epigenetic modifications, such as DNA methylation [175].

Melatonin plays an important role as a sensitizer to both irradiation and chemotherapy breast cancer treatments. Melatonin behaves as a radiosensitizer in breast cancer through the upregulation of PTEN and NME/NM23 nucleoside diphosphate kinase 1 (NME1) and the downregulation of SNAI2, HER2, and AKT. In addition, the cancer suppressor miR-34a can be enhanced by melatonin [176]. Furthermore, melatonin is a potent antioxidant, chemotherapeutic, and chemopreventive agent against breast cancer. Melatonin has potential in breast cancer treatment applications.

However, the recent research reached the surprising conclusion that the production of circulating tumor cells (CTCs) with a high metastatic tendency mainly occurs in the sleep stage and is not continuous [177]. Hematogenous dissemination of CTCs is a vital method for cancer metastasis. In breast cancer patients and mouse models, the generation of CTCs mainly occurs in the sleep stage, and these cells have a greater tendency to metastasize. Single-cell RNA sequencing analysis revealed a marked upregulation of mitotic genes, including melatonin, testosterone, and glucocorticoids during the rest period of the study [177]. Researchers have found that testosterone, glucocorticoids, and insulin play the main roles in promoting metastasis and proliferation during sleep, but the role of melatonin in this process is still not clear.

### Neurotrophic factor

Neurotrophins (NTs) are one of the most studied trophic factors and play an important role in neuronal development, survival, and apoptosis. Neurotrophic factors are growth factors and include NGF, BDNF, NT-3, and NT-4/5, and possibly NT-6 [178]. Cellular responses to NTs are elicited through two types of cell membrane receptors: the common NT receptor (NTR) p75NTR and the tyrosine kinase (Trk) receptor [15, 179]. Proneurotrophin interacts with receptor p75 (a member of the TNF receptor superfamily) or a co-receptor complex, p75, and sortilin, a transmembrane protein containing the Vps10p domain. The mature form acts by binding to specific Trks (Trk A, Trk B, and Trk C). NTs and their receptors regulate cell proliferation, migration, differentiation, survival, apoptosis, and synaptic plasticity [180, 181]. A two-protein hybrid formed by the p75NTR and Trk A monomer can enhance the specific binding affinity of NGF. However, when the homodimer formed by the polymerization of two p75 NTRs binds to NT, it can cause the opposite effect and even induce apoptosis.

Trk A receptors have higher affinity for NGF. Trk B receptors have higher affinity for BDNF and NT-4/5. Trk C receptors primarily bind to NT-3. Various receptors exist in the form of dimers, and the activation of receptors can trigger the phosphorylation of tyrosine protein kinases in the cytoplasm. By interacting with the extracellular domains of these receptors, neurotrophic factors transmit signals related to the survival and apoptosis of nerve cells to the interior of cells, thereby regulating cell behaviors. p75NTR can bind to all neurotrophic factors. In addition, there are both synergistic and antagonistic effects between p75NTR and Trk.

#### Nerve growth factor

The discovery of NGF was a milestone in the study of growth factors. NGF is necessary for the growth and survival of sympathetic and sensory neurons. Since sensory fibers supply the papilla and skin, sympathetic fibers innervate blood vessels and ducts. In particular, breast cancer cells can produce and secrete NGF, which stimulates the growth of sympathetic and sensory nerves. Likewise, there is a correlation between nerve fibers and NGF expression in breast cancer cells [8]. Axogenesis is correlated with tumor aggressiveness and is driven by NGF [182]. Axon formation can be partially reversed by blocking NGF [8]. NGF receptor (NGFR) is expressed in a variety of cancer types. BLBC shows a higher level than other subtypes [183]. NGF signaling through NGFR in CSCs may drive cancer growth *in vitro* [99].

#### Brain-derived neurotrophic factor

BDNF is the most studied and well-characterized neurotrophic factor in the CNS. In mammals, BDNF plays a role in neuronal activity, including proper growth, development, synaptic plasticity [184], neuronal differentiation [185], and neurotransmission [186], through Trk B and the low-affinity p75NTR [187]. In addition, BDNF protein is detectable in several non-neural tissues, such as endothelial cells [188] and vascular smooth muscle cells [189]. Thus, BDNF may act through angiogenesis. Recent studies have shown that cancer cell–derived BDNF promotes axon formation, which in turn promotes tumor cell invasion [99]. BDNF has been reported to be aberrantly expressed in human breast cancer, and elevated levels of BDNF are associated with poor clinical outcomes and decreased survival [190]. Knockdown of BDNF in the MDA-MB-231 and MCF-7 cell lines resulted in reduced cell proliferation and survival.

Aberrant activation of the BDNF/Trk B pathway can modulate multiple signaling pathways, including the PI3K/AKT, Jak/STAT, NF-κB, uPA/uPAR, Wnt/β-catenin and VEGF pathways, and ER receptors. BDNF can act in an autocrine or paracrine manner to increase the migratory capacity of MDA-MB-231 cells and HUVECs. BDNF-induced migration activity was blocked by inhibitors of Trk B, PI3K, and ERK in MDA-MB-231 cells. Overexpression of Trk B, but not BDNF, was significantly associated with poor survival outcomes in TNBC patients [191]. Several approaches have been developed to target this pathway, such as Trk inhibitors (AZD6918, CEP-701) and RNA interference, which can sensitize the xenograft tumors to etoposide, a topoisomerase II inhibitor [192]. One study found that estradiol (E2) treatment upregulated BDNF expression in astrocytes and later Trk B expression in breast cancer cells to promote brain metastasis of TNBC cell xenografts [193]. Interestingly, the brain metastatic process induced by E2 could be rescued by treatment with ANA-12, a BDNF/Trk B inhibitor.

However, in hormone receptor-positive mammary tumor models, BDNF overexpression in the hypothalamus of mice through a recombinant AAV vector resulted in anticancer phenotypes [194]. The above BDNF gene therapy was further reported to inhibit breast cancer cell growth by reducing inflammatory markers [194]. Therefore, BDNF might also play protumorigenic and antitumorigenic roles through different pathways in different breast cancer subtypes.

#### Neurotrophin

Various NTs have different roles in protecting neurons from apoptosis. Both NT-3 and NT-4/5 can bind to p75NTR to stimulate breast cancer cell survival [195]. NT-3 enhances breast cancer brain metastasis by promoting the transition of breast cancer cells from mesenchymal-epithelial cells to a more epithelioid phenotype and by increasing the ability of these cells to proliferate in the brain [196]. NT-4/5 is also expressed and secreted by breast cancer cells, and anti-NT-4/5 antibody treatment resulted in tumor growth inhibition characterized by increased apoptosis but no change in proliferation. NT-4/5 contributes to breast cancer cell survival and may serve as a potential target for suppressing tumor growth [197].

#### Tyrosine kinase receptors

The Trk family consists of Trk A, Trk B, and Trk C. NTs that bind to Trks are known to activate their kinase domains to trigger various downstream Ras signaling pathways involving MAPK, PI3K, and PLCγ-PKC [162]. This has primarily been established in neuronal models. Various non-neuronal cells have been reported to respond to neurotrophic factors in healthy tissues and in disease states [198]. p75NTR and Trks, two types of NTRs, have been discovered in numerous cancer types, including breast tumors [199]. Activated p75NTR, which traditionally acts as an inhibitor, induces variable effects, from inhibition to stimulation of cell proliferation in cancers, dependent on its direct or coordinated/indirect mechanism(s) of action. Trk B was reported to stimulate cell proliferation, aggressiveness, and metastasis in cancer. Anti-Trk drugs have been investigated both *in vitro* and in living mice for their effects on various cancer cells, and some are in clinical trials [199]. p75NTR and Trks were also expressed in various cancers, including breast, lung, colon-rectum, pancreas, prostate, glioblastoma, neuroblastoma, myeloma, and lymphoid tumors [199]. The potent pan-Trk inhibitor entrectinib is a promising agent targeted against many cancers including mammary carcinomas [200] Besides, a series of novel 7-azaindole-based Trk inhibitors exhibited antiangiogenic activity in breast cancer [201]. At present, the potential of LOXO-101, another anti-Trk drug, applied in soft-tissue sarcoma and colorectal cancer is in phase 2 trials [202, 203]. Other Trk inhibitors (GNF-5837, MGCD516, PLX7486, DS7486, DS6051b, and TSR-011) are underway [202].

### Netrin-1, an axon guidance factor

Netrin-1 is a laminin-related protein involved in nervous system development and regulates neurite outgrowth. In addition, netrin-1 is also involved in cell adhesion, motility, proliferation, differentiation, apoptosis, and angiogenesis. Netrin-1 and its receptors have been reported to promote tumorigenesis in a variety of cancer types [204]. By comparing 300 plasma samples from different cancer patients compared to 138 plasma samples from healthy donors, plasma netrin-1 levels were found to be significantly elevated in breast, kidney, prostate, and liver cancer and in meningiomas, pituitary adenomas, and glioblastomas. Therefore, plasma netrin-1 can be used as a diagnostic biomarker for human cancers [205]. Since netrin-1 co-localizes with endothelial CD31 in breast tumors, netrin-1 expression can be assessed via ultrasound molecular imaging (USMI), which is used to visualize vascular endothelial targets. The USMI signal of anti-netrin-1 was significantly increased in human SKBR7 mammary tumors [206].

The netrin-1 receptors, including deleted in colorectal cancer (DCC) and uncoordinated 5 homologs (UNC5H), belong to a family of dependent receptors that induce apoptosis in the absence of netrin-1, conferring the tumor suppressor activity. However, most metastatic breast cancers overexpress netrin-1. When netrin-1 was knocked down or DCC and UNC5H were overexpressed, the apoptosis of mammary metastatic tumor cell lines was increased [207]. In addition, death-associated protein kinase (DAPK)1 is a serine-threonine kinase known to transduce the proapoptotic pathway of netrin-1-dependent receptors. A considerable number of human breast tumors show a loss of DNA methylation–dependent expression of DAPK1, while inhibition of DNA methylation (such as decitabine) can restore DAPK1 expression [208]. Therefore, combining DNA methylation inhibitors with netrin-1 neutralizers may be a valuable strategy against cancer.

### Others

Neurogenic SP is an 11-amino acid peptide that belongs to the tachykinin family and is widely expressed in the central, peripheral, and enteric nervous systems of vertebrates. SP and its high-affinity receptor (neurokinin-1 receptor) have been shown to be involved in several physiological and pathophysiological processes, such as respiration, thermoregulation, immune regulation, inflammation, and tumorigenesis [209]. Both SP and neurokinin-1 are overexpressed in breast cancer and other cancer types [210]. SPs have been shown to exert anticancer functions by inducing the immune responses of T cells and NK cells [211]. However, in breast cancer, SP and its receptors have been implicated in oncogenic transformation and myeloid metastasis formation [212]. Neurokinin-1 receptor antagonists can block the mitogenic effects of SP and produce proapoptotic effects *in vitro* and *in vivo*. Therefore, the neurokinin-1 receptor is a candidate therapeutic target for cancer therapy [213]. In breast cancer cells, SP activates MMPs through neurokinin-1 receptors and turn on HER2 through Src, promoting proliferation and invasion [214, 215]. In fact, cancer cells activate autocrine circuits by releasing SP. In tumor models of breast cancer cells (MDA-MB-231 and MDA-MB-453), a neurokinin-1 antagonist (L-733,060) inhibited tumor growth and showed a synergistic effect with anti-HER2 therapy (AG825, AG1478, or lapatinib).

**Fig. 3:**
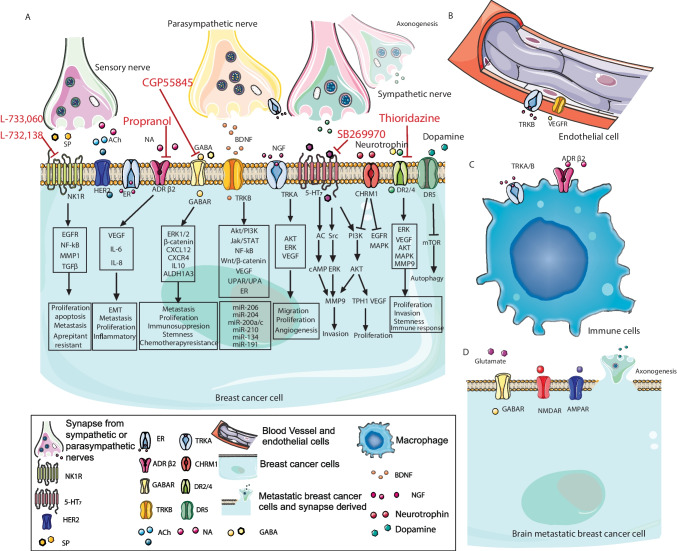
Signaling pathways of innervation and therapeutic targets in primary or metastatic breast cancer microenvironment. In the microenvironment of primary or metastatic breast cancer sites, there are complicated interactions among synapses from surrounding nerves, metastatic breast cancer cells, breast cancer cells, and immune cells. Neurotransmitter release, related receptors, and signaling pathways are included in this model. Furthermore, several effective drugs mentioned in the text are shown in red text

Neuropeptide Y enhances VEGF expression, promoting angiogenesis and breast cancer progression [216]. Neurotensin promotes metastasis by binding to neurotensin receptor 1 (NTSR1). In breast cancer, NTSR1 expression is associated with lymph node metastasis [217]. These studies demonstrate the important role of neuropeptide signaling in breast cancer metastasis (Fig. 3).

## Psychological stress promotes breast cancer progression

Chronic psychological stress is reported to be correlated with poor clinical outcomes in cancer patients [218]. The communication between neural, endocrine, and immune factors transmits various signals that induce cancer cell proliferation, invasion, metabolism reprogramming, and therapy resistance [219]. A large number of studies have demonstrated that psychosocial factors are associated with a higher incidence and lower survival in patients with different cancer types, including breast cancer [220]. Anxiety and depression are closely related to tumor development and progression due to the sustained secretion of stress-related hormones and neurotransmitters [18]. Specifically, the SNS and the HPA axis are activated when exposed to stress, releasing several neurotransmitters and hormones that promote cancer progression [221].

Preclinical breast cancer mouse models have shown that chronic restraint stress promoted breast cancer progression via catecholamine and α-AR signaling [110]. The nonselective α-adrenergic blocker phentolamine was found to inhibit tumor growth and metastasis caused by stress exposure. Interestingly, phentolamine increased primary tumor size and distant metastasis under nonstress conditions. Chronic stress–related tumor metastasis also depends on the activation of β2-AR [222]. Selective sympathetic destruction by the neurotoxin 6-hydroxydopamine (6-OHDA) decreased tumor NE concentrations by approximately 50% [223]. When exposed to stressors, tumor NE turnover was increased compared to spleen and nonstressed tumor NE turnover in mice. Ablation of sympathetic nerve function by 6-OHDA or blockade of β-adrenergic signaling by propranolol significantly inhibited stress-induced lung metastasis [224]. Depletion of circulating monocytes or lung macrophages strongly abrogated stress-induced tumor cell lung dissimilation, whereas treatment of mice with the β-adrenergic agonist isoproterenol (ISO) during the premetastatic phase promoted the infiltration of macrophages into the lung. β-Adrenergic signaling upregulates multiple genes that contribute to tumor progression and metastasis, while glucocorticoid-regulated genes can promote cancer cell survival and resistance to chemotherapy [224].

Chronic stress was reported to activate the SNS and drive malignancy. In immunodeficient murine models, chronic stress–induced E was found to promote breast cancer stem-like properties via lactate dehydrogenase A (LDHA)-dependent metabolic rewiring. Chronic stress–induced E activated LDHA to generate lactate, and the adjusted pH directed USP28-mediated deubiquitination and stabilization of MYC, promoting SLUG expression and breast cancer stem-like trait development. The LDHA-lowering agent vitamin C can be a potential approach for combating stress-associated breast cancer [225].

In a mouse model, social defeat was observed to cause anxiety-like behaviors and reduce therapeutic responses against carcinogen-induced neoplasia and transplantable tumors. Stress elevated plasma corticosterone and upregulated the expression of glucocorticoid-inducible factor Tsc22d3, which blocked type I IFN responses in dendritic cells and IFN-γ + T cell activation. Administration of a glucocorticoid receptor (GR) antagonist or DC-specific Tsc22d3 deletion reversed the negative impact of stress or glucocorticoid supplementation on therapeutic outcomes [226].

Dexamethasone (DEX) was reported to promote breast cancer cell metastasis by activating the PI3K signaling pathway and upregulating serum glucocorticoid-inducible kinase 1 (SGK1) expression. In a standard breast cancer treatment regimen with paclitaxel and DEX, DEX was found to promote lung metastasis [227]. In addition, DEX induces docetaxel and cisplatin resistance in TNBC cells by upregulating KLF5 expression, a prosurvival transcription factor, and this regulation is dependent on GR [228]. Glucocorticoid treatment facilitated GR-dependent nuclear accumulation and transcriptional activation of TEAD4, the activation of which promoted breast CSC maintenance, cell survival, metastasis, and chemoresistance both *in vitro* and *in vivo* [229].

Therefore, psychological and social factors can modulate tumor cell gene expression, reconfirming that stress promotes cancer progression by activating the SNS to release E and NE and activating the HPA axis to release cortisol. Stress reduction is an important breast cancer therapy.

Pharmacological strategies include eliminating the β2-AR signaling pathway to block the effects of E and NE [230]. Clinical trials showed that treatment with β-blockers reduced stress-related signaling, prolonging cancer patient survival [231, 232]. In addition, nonpharmacological methods such as meditation [233], yoga [234], acupuncture [235], exercise [236], support groups, counseling, natural products, and herbal compounds [237] have been reported to inhibit breast cancer [238]. Although coffee and tea can regulate the nervous system, epidemiological studies suggest that the relationship between drinking coffee or tea and breast cancer is controversial [239, 240].

Regular exercise, also called physical activity and recommended by the World Health Organization, has been observed to be associated with a 7% decrease in overall cancer risk. Among cancer types, breast cancer shows one of the strongest associations [241]. When compared with the least active women, the physically active women have a 25% average risk decrease [242]. Furthermore, increased physical activity is associated with increased overall survival of breast cancer patients [243].

All these effects are induced by regular exercise through the systemic control of energy homeostasis and metabolism, which have important effects on the inflammatory and immune mediators in breast cancer tumor microenvironment [244], including metabolic factors (IGF-1 or its binding protein IGFBP-3, insulin, glucose, C-peptide, leptin), immune and inflammatory factors (NK cell cytotoxicity, pro-inflammatory and anti-inflammatory cytokines), and oxidative stress (8-oxo-dG, F2-isoprostane) [245].

Exercise-induced TNBC prevention is mainly based on the inhibition of PI3K-AKT-mTOR signaling [244]. First, physical activity induces changes in inflammatory and immune mediators that contribute to beneficial effects on breast cancer outcomes, including tumor growth reduction, metastasis inhibition, and survival improvement. Commonly measured immune factors include splenic cell number and function, circulating inflammatory cytokines, intratumoral immune cells, and inflammatory markers. The results from preclinical and clinical studies suggest that PA exerts heterogeneous effects on inflammatory cytokines but may alter the gene expression profile and immune infiltrates in the tumor, which may result in a reduction in immunosuppressive factors [246]. Furthermore, regular exercise can upregulate oncostatin M (OSM) and myokine [247]. The exercise-inducible myokine irisin was reported to reduce the risk and improve the prognosis of breast cancer [248].

## Targeting innervation in breast cancer treatment

### Drugs targeting β-adrenergic signaling

Catecholamine-mediated activation of β-adrenergic signaling plays critical roles in cancer proliferation, invasion, metastasis, and angiogenesis. β-Blockers are commonly used in hypertension and arrhythmia treatments. In an analysis of 46,265 patients, the use of β-blockers improved cancer-specific survival [249]. β-Blocker use reduces the risk of breast cancer–related death in women with breast cancer [250] and reduces the risk of recurrence in women with breast cancer and hypertension [251]. In addition, propranolol reduces restraint stress–induced distant metastasis of orthotopic xenografts by inhibiting tumor-associated macrophage infiltration in breast cancer [81]. However, not all β-blockers are effective in breast cancer. The β1-specific blocker atenolol had no such effect [252]. In contrast, ISO, a nonselective β-AR agonist, promotes tumor invasion and metastasis [253].

The timing of β-blocker application is related to the therapeutic efficiency. Patients who used β-blockers before cancer diagnosis had a 57% lower risk of metastasis and a 71% reduction in breast cancer mortality after 10 years. Tumor recurrence was significantly reduced [104]. The use of β-blockers at the start of neoadjuvant therapy was associated with slightly better recurrence-free survival but not overall survival [254, 255]. Preoperative use of β-blockers (propranolol) is associated with decreased EMT and decreased prometastatic/proinflammatory transcription factor activity in early breast cancer [255].

Concomitant preoperative use of β-blockers (propranolol) reduces mesenchymal gene expression in primary early breast cancer tumors. Nonselective β-blockers employed in patients with early breast cancer may lead to reduced tumor proliferation [256]. In general, β-blockers may improve immune surveillance a few days before surgery [257]. This may be correlated with increased catecholamine-mediated immunosuppressive activity after surgery, possibly attributable to psychological distress. In addition, pharmacologic (propranolol) and genetic (β2-AR knockout) inhibition reduced tumor growth implanted under chronic cold stress [114]. Therefore, the introduction of β-blockers in breast cancer treatment requires strict control of disease progression, treatment timing, and basic health regulation of patients to achieve the best results (Table 3 and Fig. 3) [24].

**Table 3:** Therapeutic drugs targeting innervation

Properties	Drug name	Drug target	Functions	Reference
Antagonists or inhibitors	Propranolol	β1/β2-AR	Be associated with T4 or N2/N3/M1 tumor incidence, cancer-specific mortality or RFS; decrease EMT, reduce metastatic; improve cancer-specific survival; reduce recurrence	[24, 27, 77, 214]
	Atenolol	β1-AR	No function	[240]
	AZD6918, CEP-701	Trk B	Prolong survival outcomes	[186]
	CGP55845	GABABR	Reduce cell migration and invasion *in vitro* and metastasis *in vivo*	[249]
	SB269970	5-HT7 receptor	Inhibit cell proliferation, migration, and invasion	[85]
	SSRIs	5-HT transporter, 5-HTR2B	Control tumor growth combined with chemotherapeutic drugs; increased breast cancer mortality	[127, 128]
	GYKI52466	Glutamate/AMPA	Decrease motility and invasive growth; inhibit cancer cell proliferation and invasion	[114]
	MK-801 or dizocilpine	NMDAR	Inhibit cancer cell proliferation and invasion	[157]
	L-733,060	Neurokinin-1	Inhibit tumor growth and synergistic with anti-HER2 therapy	[203–205]
	Trifluoperazine and thioridazine	D2R	Reduces cancer stem cell proliferation and invasiveness	[117]
Agonists or activators	Isoproterenol	Non-selective β-AR	Sympathetic activation and promotes tumor invasion and metastasis	[214]
	Nicotine	α9-nAChR	Promotes MCF-7 and MDA-MB-231 breast cancer cell migration through EMT	[106, 107]
	Propofol	GABA-A receptors	Increases breast cancer cell migration	[246–248]
	Baclofen	GABABR	Promoted 4T1 cell invasion, migration, and metastasis	[249]

*T* tumor, *N* node involvement, *M* metastasis, *RFS* relapse-free survival

### Drugs targeting GABA and the 5-HT transporter

The use of the intravenous anesthetic propofol in breast cancer surgery is reported to be significantly associated with breast cancer prognosis by both promoting and inhibiting breast cancer growth and metastasis [258]. On the one hand, propofol was observed to increase MDA-MB-468 cell migration [259] by increasing intracellular calcium and altering the actin cytoskeleton organization [260]. One possible explanation is that propofol increases breast cancer cell migration by activating GABA-A receptors, a process that increases intracellular reorganization of L-type calcium channels and the calcium-related actin cytoskeleton [260]. Consistently, the GABABR agonist baclofen significantly promoted 4T1 cell migration and invasion, which was attenuated by the GABABR antagonist CGP55845 [261]. In addition, the proliferation and migration of MDA-MB-231 cells were increased by propofol treatment, which was explained by the correlation with upregulation of nuclear factor E2-related factor 2 and downregulation of p53 expression [262]. On the other hand, propofol significantly suppressed the migration and invasion of MDA-MB-231 cells [263] by reducing MMP-2 and MMP-9 expression and decreasing neuroepithelial cell–transforming gene 1 (NET1) [264], which are important in promoting migration. In addition, the propofol also downregulated miR-24 and increased p27 expression and cleaved caspase-3 expression in MDA-MB-435 cells, leading to induced cell death [265]. In another study, MDA-MB-231 cells were incubated with serum from patients after propofol/paravertebral block anesthesia group, resulting in decreased viability due to increased apoptosis compared to that of cells incubated with serum from patients receiving sevoflurane/opioid anesthesia [266]. Similarly, when ER + /PR + breast cancer cells (HCC1500) were co-cultured with NK cells obtained from healthy donors in the presence of the above serum, HCC1500 cells underwent apoptosis. Concomitantly, CD107a is upregulated in stimulated NK cells and results in NK cell–mediated lysis of target cells [267]. The inconsistent effects of propofol in breast cancer cells were correlated with different breast cancer subtypes and the dosage and exposure time. This might be explained by the fact that when combined with paravertebral block, propofol causes serum composition changes, affecting breast cancer cell behavior and NK cell activity [258].

However, there are uncertain results concerning the application of SSRIs in breast cancer treatments. Since nearly 50% of breast cancer patients suffer from depression or anxiety, first-line antidepression drugs have been implicated in breast cancer progression through increased prolactin levels and tamoxifen metabolism inhibition. SSRIs and serotonin receptor inhibitors were found to inhibit breast cancer–initiating cell activity in mice [134]. Sertraline (Zoloft) promoted the efficiency of docetaxel in controlling breast tumor growth [134] and contributed to the relief of hot flashes in breast cancer survivors taking tamoxifen and to controlling the accompanying depression [268]. However, selective SSRI use, either before or after breast cancer diagnosis, was observed to be co-related with an increase in overall mortality [135].

### Nerve-specific blocking virus

It is well known that sympathetic nerves are cancer-promoting in breast cancer, while parasympathetic/vagal nerves play a tumor-suppressing role in breast cancer. These neural effects may be mediated by β-adrenergic or muscarinic receptors and may be explained by changes in cancer cell behavior, angiogenesis, tumor-associated macrophages, and adaptive antitumor immunity. A recently developed viral vector-based genetic localized neural engineering technique is a powerful approach to selectively manipulate specific types of nerve fibers that innervate the cancer microenvironment, thereby inhibiting cancer progression [16]. The technology will enable the creation of “cancer neurotherapies” that are individually tailored to different cancer types [24]. AAV vector-based gene deletion or local tumor sympathetic nerve stimulation greatly reduced or increased primary tumor growth and distant metastasis of orthotopic xenografts and chemo-induced tumors [16] and was more efficient than the β-blockers because of the immune-suppression function [24]. Thus, cancer-specific and nerve-specific manner would be more accurate while pharmacological blockade would target different organs with α- or β-adrenergic receptor expression. In detail, tumor-specific sympathetic denervation downregulated the expression of immune checkpoint molecules (PD-1, PD-L1, and FOXP3) while genetically simulated parasympathetic innervation decreased PD-1 and PD-L1 expression in immune-competent breast cancer models. These neural effects may be mediated by the M1 Chrm, since the M1-specific antagonist pirenzepine decreased these effects [16].

### Relieve pressure strategies

The development of breast cancer is associated with a variety of factors, including stress, anxiety, depression, sleep disturbance, and fatigue. Stress promotes cancer progression by activating the SNS to release E and NE and by activating the HPA axis to release cortisol. The National Center for Complementary and Alternative Medicine, an initiative of the National Institutes of Health (NIH) [269], recommends the use of health care, including mind–body medicine, nutritional supplements, herbal products, exercise, and other energy-based techniques and practice programs, for disease treatment. A European study that included 11 countries reported complementary and alternative medicine (CAM) use in 44.7% of breast cancer patients [270], resulting in high levels of satisfaction, while only 6.5% of the women reported no benefits. In a US study, 86.1% of patients used CAM after breast cancer diagnosis: 47.5% used botanical supplements, 47.2% used other natural products, 28.8% used special diets, 64.2% used mind–body therapy, and 26.5% used body/energy/other treatments [271]. Other CAMs used by breast cancer patients include spiritual methods such as prayer, meditation, and psychotherapy [272]. Spiritual and religious practice has been one of the coping strategies used by breast cancer patients [273]. After assessing the quality of related reviews of CAM studies, CAM could be concluded to be helpful for treating specific symptoms related in breast cancer patients [274] and thus should be considered before clinical treatment. In addition, methods such as therapeutic massage in breast cancer patients have shown beneficial effects on the neuroendocrine and immune systems, reducing anxiety levels, depression, anger, fear, and stress and improving NK and lymphocyte counts.

In the past decade, the development of neuro-oncology has achieved milestones, and numerous studies from different aspects have verified that the nervous system plays an active role in the occurrence and development of breast cancer. Furthermore, ablation of specific nerve types (parasympathetic, sympathetic, or sensory) can eliminate tumor growth in a tissue-specific manner, showing a promising prospective role in breast cancer treatment.

## Conclusions and perspectives

Neuro-oncology has undoubtedly developed into an independent emerging discipline, with several ensured conclusions:

1. *Peripheral nerves and solid tumors support each other’s growth directly or indirectly*. It is important to elucidate the signaling pathways initiated by neurotransmitters, neuropeptides, and their associated receptors. Global characterization of the neurotransmitters or neuropeptides present in the tumor microenvironment will provide insight into the true biological impact of neuronal organization on tumor progression. Cancer cells can take advantage of factors released by nerve fibers to create a microenvironment for cancer cell survival and proliferation. Cancer cells can also behave like nerves in a microenvironment stimulated by nerves and their neurotransmitters or neuropeptides, which can be both released from both nerves in the microenvironment and cancer cells as well. In return, the secretion of neurotrophic factors and axon-guiding molecules by breast cancer cells stimulates synapse formation, and newly formed adrenergic nerve fibers promote tumor growth.
2. *Breast cancer neural invasion not only leads to breast cancer treatment resistance but also promotes distant metastasis and increases the mortality of breast cancer patients*. According to a large number of clinical and preclinical studies, innervation in breast cancer alters gene expression, which is co-related with chemotherapy resistance, tumor angiogenesis, lymphangiogenesis, and brain metastasis. In addition, innervation can also modulate the immune response mediated by cytokines and immunocytes in the breast cancer microenvironment.
3. *Furthermore, multiple clinical strategies such as pharmacological and genetic approaches could be applied to block neuronal signaling pathways in the TME, showing a promising therapeutic potential for the treatment of breast cancer*. The ensured pharmacological drugs are mainly β-blockers. Genetic technology specifically aimed at different nerve types is still under development.
4. *The SNS and the HPA axis are activated by stress to promote breast cancer progression*. Nonpharmacological methods to relieve pressure, such as meditation, yoga, acupuncture, physical activity, support groups, counseling, natural products, and herbal compounds, would benefit breast cancer patients.

Therefore, in-depth exploration of the mechanisms of neural infiltration and its important contribution to the synergistic network among tumors, the immune system, and endocrine cells is conducive to targeted intervention. Further understanding of the network would overcome the unfavorable factors in breast cancer treatment and improve the survival of breast cancer patients. Currently, the most ensured and effective treatments that are beneficial in breast cancer patients, aside from the standard regimen, are pharmacological methods to reduce stress, such as taking β-blockers, and other methods such as exercising and relaxing. Therefore, understanding the role of nerves played in breast cancers is promising for future cancer therapies, and nerves could be valuable targets alone or in combination with the currently available standard therapeutic regimens.

However, further studies are required to solve the unknown aspects of current studies. The exact role of each nerve in the complex neuronal network and their interaction with each other in the face of the changeable breast cancer environment according to different development and treatment stages and even the psychological stress levels in each patient need to be clearly elucidated. Although the immune checkpoint molecules can be regulated by innervation or denervation through pharmacological or genetic techniques in preclinical animals, they need to be improved before being applied safely and efficiently in clinical managements.

In addition, the nervous system is quite sophisticated, and mature neuron’ culture *in vitro* is challenging. The development of appropriate models to investigate the interaction of nerves and tumor cells *in vitro* and *in vivo* is currently limited. Researchers usually investigate nerve roles through mouse models that are genetically edited and transplanted with tumors with or without metastatic sites. Physical activity and CAM strategies can be observed in human beings.

Furthermore, although it has been confirmed that physical and relaxing activity can benefit cancer patient survival, it is significant to elucidate the detailed mechanisms and the specific way the highest efficiency can be obtained when combined with clinical treatments. In addition, clinicians should also pay attention to the CAM employed by patients before and after regimen therapies, and it would be helpful to make standard guidelines as supplementation for clinical therapies.

Therefore, a good and appropriate mouse model or special clinical cases with innervation in the primary site or with brain metastasis would be a good opportunity to discover more about the neuro-malignancy circuit.

## Data Availability

There is no data relevant to be presented in this work.

## Abbreviations

5-FU: 5-Fluorouracil
5-HT: 5-Hydroxytryptamine
6-OHDA: Neurotoxin 6-hydroxydopamine
AAV: Adeno-associated virus
ACh: Acetylcholine
AChE: Acetylcholinesterase
ALDH: Aldehyde dehydrogenase
AMPA: α-Amino-3-hydroxy-5-methyl-4-isooxazole propionate
AR: Adrenergic receptor
ATP: Adenosine triphosphate
BBB: Blood-brain barrier
BCBM: Breast cancer brain metastasis
bChE: Butyrylcholinesterase
BDNF: Brain-derived neurotrophic factor
BLBC: Basal-like breast cancer
BMOR: Brain metastasis oncogenic lncRNA
CAM: Complementary and alternative medicine
cAMP: Cyclic adenosine monophosphate
Chrm1: Cholinergic receptor 1
CIBP: Cancer-induced bone pain
CNS: Central nervous system
CSC: Cancer stem cell
CTC: Circulating tumor cell
CXCL: Chemokine ligand
DAPK: Death-associated protein kinase
DCC: Deleted in colorectal cancer
DEX: Dexamethasone
DR: Dopaminergic receptor
DRG: Dorsal root ganglia
E: Epinephrine
E2: Estradiol
EMT: Epithelial-to-mesenchymal transition
ER: Estrogen receptor
ERK: Extracellular signal–regulated kinase
GABA: Gamma-aminobutyric acid
GABRP: Pi subunit of the GABA-A receptor
GAD: Glutamate decarboxylase
GEO: Gene Expression Omnibus
GPCR: G protein–coupled receptor
GR: Glucocorticoid receptor
HER2: Human epidermal growth factor receptor 2
HPA: Hypothalamic-pituitary-adrenal
HTR1A: 5-HT receptor 1A
IFN: Interferon
IGF: Insulin-like growth factor
iGluR: Ionotropic glutamate receptor
IL: Interleukin
ISO: Isoproterenol
LDHA: Lactate dehydrogenase A
mAChR: Muscarinic acetylcholine receptor
MAOA: Monoamine oxidase A
MAPK: Mitogen-activated protein kinase
METABRIC: Molecular Taxonomy of Breast Cancer International Consortium
MFS: Metastasis-free survival
mGluR1/Grm1: Metabotropic glutamate receptor 1
mTOR: Mechanistic target of rapamycin
nAChR: Nicotinic acetylcholine receptor
NE: Norepinephrine
NET: Neuroepithelial cell–transforming gene
NGF: Nerve growth factor
NK: Natural killer
NMDAR: *N*-Methyl-d-aspartate receptor
NT: Neurotrophin
NTR: Neurotrophin receptor
NTSR1: Neurotensin receptor 1
Oct-4: Octamer-binding transcription factor 4
OSM: Oncostatin M
PD-1: Programmed death 1
PD-L1: Programmed death ligand 1
PI3K: Phosphoinositide 3-kinase
PKA: Protein kinase A
PNS: Peripheral nervous system
PTEN: Phosphatase and tensin homolog
SGK1: Serum glucocorticoid-inducible kinase 1
SNS: Sympathetic nervous system
SP: Substance P
SSRI: Selective serotonin reuptake inhibitor
TCGA: The Cancer Genome Atlas
TGF-β: Transforming growth factor beta
TME: Tumor microenvironment
TNBC: Triple-negative breast cancer
TNF: Tumor necrosis factor
TPH: Tryptophan hydroxylase
Trk: Tyrosine kinase
TRPV1: Transient receptor potential vanilloid 1
UNC5H: Uncoordinated 5 homolog
USMI: Ultrasound molecular imaging
VEGF: Vascular endothelial growth factor
α7nAChR: α-7-Nicotinic acetylcholine receptor
